# Physiological and immunological responses to *Culicoides sonorensis* blood-feeding: a murine model

**DOI:** 10.1186/s13071-018-2935-0

**Published:** 2018-06-20

**Authors:** Christopher J. Lehiy, Lindsey M. Reister-Hendricks, Mark G. Ruder, D. Scott McVey, Barbara S. Drolet

**Affiliations:** 1 Arthropod-Borne Animal Diseases Research Unit, Agricultural Research Service, US Department of Agriculture, Manhattan, KS 66502 USA; 2 Southeastern Cooperative Wildlife Disease Study, College of Veterinary Medicine, University of Georgia, Athens, GA 30602 USA

**Keywords:** *Culicoides sonorensis*, Midge, Mice, Saliva, Bite, Innate immunity

## Abstract

**Background:**

Hematophagous *Culicoides* spp. biting midges are of great agricultural importance as livestock, equine, and wildlife pests and as vectors of the orbiviruses bluetongue, epizootic hemorrhagic disease and African horse sickness. To obtain a blood meal, midges deposit saliva containing allergens, proteases, and anti-hemostatic factors, into the dermis to facilitate feeding. Infected midges deposit virus along with the myriad of salivary proteins during feeding. The extreme efficiency with which midges are able to transmit orbiviruses is not clearly understood, as much is still unknown about the physiological trauma of the bite and immune responses to saliva deposited during feeding. Of particular interest are the first few hours and days after the bite; a critical time period for any midge-transmitted virus to quickly establish a localized infection and disseminate, while avoiding the hosts’ immune responses.

**Results:**

A mouse-midge feeding model using colonized *Culicoides sonorensis* midges was used to characterize innate mammalian immune responses to blood-feeding. Histological analysis of skin, and cellular and cytokine profiles of draining lymph nodes show *Culicoides* midge feeding elicited a potent pro-inflammatory Th-mediated cellular response with significant mast cell activation, subcutaneous hematomas, hypodermal edema and dermal capillary vasodilation, and rapid infiltration of leukocytes to the bite sites. Mast cell degranulation, triggered by bite trauma and specifically by midge saliva, was key to physiological and immunological responses and the ability of midges to feed to repletion.

**Conclusions:**

Midge feeding causes physiological and immunological responses that would be highly favorable for rapid infection and systemic dissemination orbiviruses if delivered during blood-feeding. Recruitment of leukocytic cells to bitten skin brings susceptible cell populations in proximity of deposited virus within hours of feeding. Infected cells would drain to lymph nodes, which become hyperplastic in response to saliva, and result in robust viral replication in expanding cell populations and dissemination *via* the lymph system. Additionally, saliva-induced vasodilation and direct breaches in dermal capillaries by biting mouthparts exposes susceptible vascular endothelial cells, thereby providing immediate sites of virus replication and a dissemination route *via* the circulatory system. This research provides insights into the efficiency of *Culicoides* midges as orbivirus vectors.

## Background

*Culicoides* comprise a diverse genus of hematophagous insects within the biting midge family Ceratopogonidae. In North America, one of the most abundant *Culicoides* species is *C. sonorensis* (Wirth & Jones) with a geographical range extending from the Atlantic to the Pacific coasts and from Florida to Canada [1, 2]. *Culicoides sonorensis* midges preferentially feed on domestic and wild ruminants and horses but are known opportunistic feeders of a variety of wildlife [3, 4]. Feeding in swarms, their attack rates have been reported as high as 110 per minute with collections of as many as 281 fed females from a single animal in the field after only a 10 min exposure period [5, 6]. Thus, the potential impact of such intense feeding on mammalian immune responses could be substantial. Additionally, unlike vessel-feeding mosquitos, *Culicoides* are “pool” feeders causing significant mechanical damage to the dermis, similar to *Simulium* (black flies), Tabanidae (horse flies), Psychodidae (sand flies) and Ixodidae (ticks) [7]. Specialized mouth parts pierce through the epidermis inducing dermal vascular damage and hematomas. Saliva containing anti-hemostatic factors, protease inhibitors, and immune modulatory proteins [8–10] is deposited to facilitate feeding. *Culicoides* spp. have been shown to transmit a number of animal-associated pathogens in their saliva including orbiviruses: bluetongue virus (BTV) [11], epizootic hemorrhagic disease virus (EHDV) [12–14] and African horse sickness virus (AHS) [15]; rhabdoviruses (vesicular stomatitis virus [16–18] and bovine ephemeral fever virus [19]); and bunyaviruses (Oropouche virus [20] and Schmallenberg virus [21]). The effects of blood-feeding on the efficiency of viral transmission and the ability of these arboviruses to establish infection before being cleared by the mammalian immune system is not clearly understood.

Evidence of vector-enhanced transmission has been shown in previous studies where a single BTV infected midge was capable of inducing viremia, clinical signs, and seroconversion of a susceptible host [22], whereas reproducible needle inoculation infections can require up to 7 logs of cell culture-derived virus [23]. Sheep exposed to naïve *Culicoides*, followed directly by intradermal injection of virus, had higher viremia and more pronounced clinical signs compared to intradermal injection alone, despite the fact that injections introduced at least 10 times more virus than midge feeding [24]. Furthermore, sheep inoculated with BTV in combination with *Culicoides* salivary proteins had more severe and varied clinical signs for three times longer than sheep receiving the viral inocula alone [25].

Insight of allergenic properties of *Culicoides* saliva has been obtained in the study of sweet itch, a painful, intensely pruritic dermatitis due to immunoglobulin E (IgE)-mediated hypersensitivity response primarily in Icelandic horses and Awassi sheep [26–28]. Horses with insect bite hypersensitivity (IBH), when exposed to *Culicoides*, develop a robust T-helper type 2 (Th2) lymphocyte response, triggering B-cell proliferation and class switching towards the production of IgE [29]. Subsequent work with peripheral blood mononuclear cells obtained from IBH-susceptible and resistant horses demonstrated that stimulation with *Culicoides* whole body extracts induced high levels of interleukin-4 (IL-4) [30]. Subsequent research refined the causative agent for this IBH response from whole body extracts to several proteins within *Culicoides* saliva [31, 32].

In spite of these inroads in understanding *Culicoides* as a vector of pathogens and the causative agent for some allergic responses, little is known about the local physiological effects of *Culicoides* feeding on host skin, particularly regarding the cellular immune responses elicited in the hours and days directly after a blood meal has been taken. We used a mouse-midge feeding model to examine skin and draining lymph nodes proximal to *C. sonorensis* feeding sites for changes in dermal architecture, immune cell populations, and in cytokine production. Here, we show that *Culicoides* midge feeding, and deposition of saliva into the dermis, potentiates a mast cell degranulation-dependent Th-2-mediated response. This leads to a rapid infiltration and proliferation of CD16/32+ cells; known targets of orbivirus infection [33, 34]. Additionally, both the mechanical disruption of dermal vasculature, and the vasodilatory effects of the saliva, result in exposure of endothelial cells, known targets of orbivirus infection [35]. Overall, our results suggest that early immune responses to *Culicoides* midge feeding likely create a very hospitable environment for initial local infection by midge-vectored orbiviruses, as well as facilitate efficient blood and lymph systemic viral dissemination.

## Methods

### Insects

Four-day-old adult female *Culicoides sonorensis* midges [36] from the Arthropod-Borne Animal Diseases Research Unit (ABADRU, Manhattan, KS, USA) colony were used in each of the feeding experiments. Prior to use, insects were maintained at 27 °C on a 10% sucrose diet, *ad libitum*.

### Mice

Specific pathogen-free female BALB/c mice (8–10 week-old), mast cell-sufficient (WBB6F1-^+/+^) and mast cell-deficient (WBB6F1-^W/Wv^) mice (10 weeks-old) were purchased from The Jackson Laboratory (Sacramento, CA, USA) and housed in the ABADRU animal maintenance facility to acclimate for one week to reduce stress and ensure health. Six mice were used per treatment group for each experiment. Negative control (naïve) mice had no exposure to midge feeding. All mice, including negative controls, were anesthetized with a ketamine (80 mg/kg) and xylazine (5 mg/kg) cocktail. To mitigate effects of bacterial flora and facilitate the midge’s access to skin, abdomens were wiped with ethanol-soaked gauze pads, allowed to dry completely, hair removed by shaving followed by application of a depilatory cream, and again thoroughly wiped with ethanol-soaked gauze pads. Mouse abdomens were positioned over the mesh top of a feeding cage containing approximately 300 adult female midges. Cages were placed in a low light environment and midges were allowed to feed for 30 min. Midges were placed in a -20 °C freezer for a minimum of 3 h and the number of fully and partially fed females counted under a Nikon SMZ1500 stereozoom microscope (Nikon, St. Louis, MO, USA). BALB/c mice were euthanized at 1, 2 or 3 days post-feeding (dpf) and WBB6F1 mice were euthanized at 3 h post-feeding (hpf) with a ketamine (100 mg/kg) and xylazine (10 mg/kg) cocktail. Excised abdominal skin was immediately visualized with transmitted and/or reflected LED illumination on a Nikon SMZ1500 stereozoom microscope (Nikon).

### Generation of connective tissue mast cells from mouse bone marrow

Bone marrow-derived mast cells (BMMCs) were generated as previously described [37, 38]. Briefly, cells were extracted from mast cell-sufficient mice (WBB6F1-^+/+^) into RPMI 1640 media (Invitrogen, Grand Island, NY, USA) containing 10% fetal bovine serum and a 1X penicillin-streptomycin-Fungizone cocktail (Invitrogen). After two changes of media to remove debris and non-adherent cells daily for 3 days, recombinant Interleukin-3 (rIL-3; 3 U/ml) (EBiosciences, San Diego, CA, USA) was added to the media and the cells were incubated at 37 °C with 5% CO_2_. After 3 weeks, the cells were divided into two aliquots and assayed for mast cell lineage using the c-KIT (CD117) rat anti-mouse antibody-FITC conjugated or Fc-epsilon Receptor I (FcεRI) rat anti-mouse antibody-FITC conjugated antibodies with flow cytometry (EBiosciences). At this step, 89% of the cells were identified as BMMC lineage.

Connective tissue mast cells (CTMCs) were generated as previously described [39] with minor modifications. Briefly BMMCs were co-cultured with 3T6 fibroblast cells (CCL-96; ATCC, Manassas, VA, USA) supplemented with rIL-3 (10 U/ml) and recombinant stem cell factor (10 U/ml) (EBiosciences) for 3 weeks. To test for CTMC differentiation, cells were treated with compound 48/80 (Sigma-Aldrich, St. Louis, MO, USA) and tested for β-hexosaminidase secretion using the Mouse Beta-hexosaminidase-A ELISA kit (Novaprotein, Summit, NJ, USA). Data were collected using the Gen 5 Microplate Reader Software on the BioTek Synergy H4 plate reader (BioTek Instruments Inc., VT, USA).

### *Culicoides* saliva collection

Saliva was collected and pooled from the *C. sonorensis* colony using an artificial membrane feeder system, as previously described [40]. Briefly, saliva was collected onto 0.22 μm hydrophilic membrane discs (Millipore, Billerica, MA, USA) from 3- to 14-day-old adult female midges. Discs were placed into an elution solution containing phosphate buffered saline (PBS) pH 7.4 supplemented with 1 mM 3-[(3-Cholamidopropyl) dimethylammonio]-1-propanesulfonate (CHAPS; Sigma-Aldrich) and stored at 4 °C for up to two weeks. Salivary proteins in the eluent were concentrated using an Amicon Ultra-15 Centrifugal Filter containing an Ultracel-3 membrane (Millipore) with a molecular weight cut off of 3 kilodaltons and stored at -80 °C. Protein concentrations were determined with a Nanophotometer (Implen, Westlake Village, CA, USA).

### Mast cell degranulation in response to *Culicoides* salivary proteins

Differentiated CTMC cells, as well as 3T6 fibroblasts that had not been co-cultured with BMMC cells, were placed in a 12 well plate format at 2.5 × 10^5^ cells per ml and allowed to reach 80% confluence overnight. Media was removed from the cells and washed 3 times with RPMI 1640 (Invitrogen) lacking FBS or recombinant cytokines. Various amounts ranging between 100 ng to 1 μg of *C. sonorensis* saliva collected using an artificial membrane were added to 1 ml of RPMI 1640, mixed and added to each well in triplicate. For negative (0 ng) and positive controls, PBS or 1 μl compound 48/80 (Sigma-Aldrich) was added to 1 ml of RPMI 1640 and added to cells, respectively. After 1 h, the supernatant was carefully removed from each well and centrifuged at 300× *g* for 10 min to remove cellular debris. Each supernatant was then assayed for β-hexosaminidase levels using the Mouse Beta-hexosaminidase A ELISA kit (Novaprotein). Results were analyzed by 2-way ANOVA with Sidak’s multiple comparisons test.

### Generation of CTMC-reconstituted WBB6F1-^W/Wv^ –R mice

Mast cell-deficient (WBB6F1-^W/Wv^), 10-week-old mice were anesthetized as previously described and carefully injected between the skin and peritoneal layer of the belly area with 2.5 × 10^6^ CTMCs in 50 μl of sterile PBS. The CTMC-mast cell reconstituted mice (WBB6F1-^W/Wv^ -R) were allowed to recover for 2 days and then exposed to feeding *C. sonorensis* as described above. Statistical significance for midge feeding rates was determined by multiple t-tests with two-stage linear step-up procedure of Benjamini et al. for controlling the false discovery rate [41].

### Inguinal and popliteal lymph node cytology and immunophenotyping

Inguinal and popliteal lymph node pairs were excised from mice euthanized at 1, 2 and 3 dpf with the surrounding fat pad carefully separated from the nodes. Node pairs were immediately suspended in ice cold PBS and made into single cell suspensions by macerating through a 100 μm cell strainer (BD Bioscience, San Jose, CA, USA). The cells were centrifuged 500× *g* for 1 min and washed 3 times with 1 ml volumes of ice cold PBS. Cell suspension samples to be counted were mixed 1:1 with 0.4% solution of trypan blue (Thermo-Fisher Scientific, Waltham, MA, USA) and manually counted with a hemocytometer for each node pair. Statistical significance was determined by multiple t-tests with two-stage linear step-up procedure [41].

Cell phenotype identification was completed on an Accuri C6 flow cytometer and collected with CFlow Plus software (Accuri Cytometers, MI, USA) following the protocols previously described for lymph node manipulation [42]. Briefly, 6 × 10^5^ cells from each node pair were resuspended in 600 μl PBS with 0.1% (v/v) Tween 20 and 1% bovine serum albumin (Sigma-Aldrich). Cells were divided into six aliquots and incubated in the dark on ice for 1 h unlabeled or with 0.1 μg of each of the following directly conjugated primary antibody pairs from BD Bioscience unless otherwise noted: T helper cell- CD-3 FITC/CD-4 PE, T cytotoxic cell- CD-3 FITC/CD-8a PE, Macrophage- CD-11b FITC/ F4/80-PE (Biolegend, San Diego, CA, USA), and B cells- B220 FITC/CD-19 PE. After incubation, cells were washed three times and resuspended in 200 μl ice cold PBS prior to analysis. Gating strategies were applied using a forward scatter (FSC)/side scatter (SSC) plot to remove debris and analyze complexity of the collected samples. Each sample was gated further to determine antibody labeling and analyzed as a final percentage of the total cell population collected. Statistical significance was determined with multiple t-tests with two-stage linear step-up procedure [41].

### Inguinal lymph node cytokine quantification

Remaining inguinal lymph node cells from 1, 2 and 3 dpf groups were pooled per group and resuspended in 1 ml of RLT buffer (Qiagen, Valencia, CA, USA). The resuspended cells were then frozen at a minimum of 1 day at -80 °C prior to RNA extraction. Upon thawing, the tubes were centrifuged at 1500× g for 5 min to remove debris and transferred to a clean tube. Total RNA isolation was done with the RNA Easy kit (Qiagen) with DNase digestion following manufacturer’s protocol, checked for purity using 260/280 nm absorbance ratios, and quantified using a Nanophotometer (Implen). Quantification of mouse cytokines (TNF-α, IFN-γ, IL-2, -4, -5, -10, -23) from the pooled total RNA samples was done in triplicate, with β-actin internal and no-RT controls, using the PrimeTime qPCR probe-based assays from Integrated DNA technologies (IDT, Coralville, IA, USA) and the SuperScript® III Platinum® One-Step qRT-PCR Kit (Invitrogen) following the manufacturer’s protocol. Beta Actin was used as an internal control. Cytokine expression level differences compared to naïve controls are reported as 2^-ΔΔC^_T_ corrected fold difference [43].

### Cytokine quantification from excised skin

Excised abdominal skin (25–30 mg) from naïve or exposed wild type mast cell-sufficient (WBB6F1-^+/+^), mast cell-deficient (WBB6F1-^W/Wv^), and CTMC-reconstituted (WBB6F1-^W/Wv^ -R) mice was placed in 1ml of RLT buffer (Qiagen) and macerated with an Omni flex tip homogenizer (Omni international, Kennesaw, GA, USA) on high speed for 2 min on ice. Samples were then centrifuged at 1500× *g* to remove debris and cleared supernatants transferred to a new tube. Total RNA was extracted with an RNA Easy kit (Qiagen) following manufacturer’s protocol and quantified using a Nanophotometer (Implen). Mouse cytokines [granulocyte-macrophage colony-stimulating factor (GM-CSF), IL-12, 17, chemokine C-X-C motif ligand 2 (CXCL2), IFN-α/β receptor α chain (IFNAR)] were quantified using PrimeTime qPCR Assays (IDT) and the SuperScript® III Platinum® One-Step qRT-PCR Kit (Invitrogen) following the manufacturer’s protocol. Beta Actin was used as an internal control. Additionally, wild type (WBB6F1-^+/+^) and CTMC-reconstituted (WBB6F1-^W/Wv^ -R) groups were compared to the mast cell-deficient (WBB6F1-^W/Wv^) group by Holm-Sidak t-test for multiple comparisons.

### Hematoxylin and eosin staining, anti-CD16/32+ labeling, and chloroacetate esterase staining of mouse skin

Excised abdominal skin was secured to nylon mesh and submerged in 10% buffered formalin for a minimum of 3 days. After skin was embedded in paraffin, 5 μm cross sections were cut. Sections were deparaffinized and stained with Hematoxylin 560, Blue Buffer 8, Define, and Alcoholic Eosin Y 515 (H&E; Leica, Buffalo Grove, IL, USA) following the manufacturer’s protocol. For labeling CD16/32+ cells, deparaffinized tissues were blocked for 1 h in 6% casein solution, rinsed twice with PBS, incubated for 1 h with 1:400 dilution of FITC Rat anti-mouse CD16/CD32 antibody (BD Biosciences) in 5 ml PBS, rinsed twice in PBS and stained for 5 min in a 0.5% methyl green solution (Electron Microscopy Sciences, Hatfield, PA, USA). For chloroacetate esterase staining, sections were deparaffinized and washed for 10 min with distilled water and stained with the Napthol AS-D Chloroacetate Esterase kit (Sigma-Aldrich) as directed. A minimum of 10 sections from each mouse were stained and blinded for examination. Bright-field microscopy for H&E sections, mast cell activity and FITC fluorescence microscopy was done with Nikon 80i (Nikon). Representative images were taken using Leica DFC 7000T camera (Leica Biosystems, Buffalo Grove, IL, USA), using identical exposure settings across all treatment and control samples. Images were processed in Adobe Photoshop (Adobe Systems, San Jose, CA, USA) using the auto levels tool across all treatment and control samples.

## Results

### Cellular responses to *C. sonorensis* feeding

**Fig. 1:**
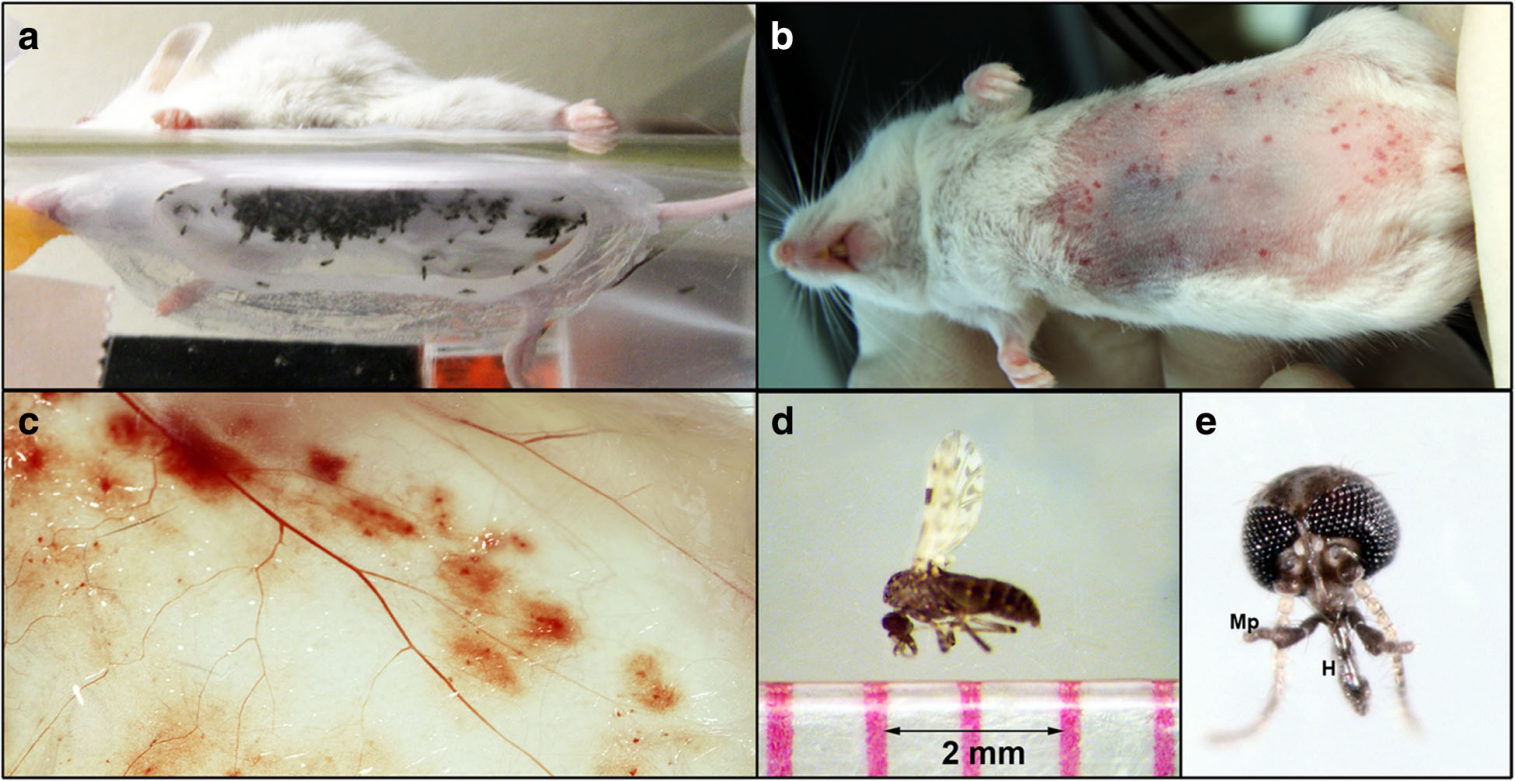
*Culicoides sonorensis* midge feeding on BALB/c mice. **a** 10-week-old mouse on a feeding cage containing 4-day-old female *C. sonorensis* midges. **b** Mouse abdomen showing multiple petechial hemorrhages associated with bite sites immediately after midge feeding. **c** Subcutaneous view of skin immediately after midge feeding (7.5×). **d** Size of adult female *C. sonorensis* biting midge. **e** *Culicoides sonorensis* head with splayed biting mouthparts hypopharynx (H) and maxillary palps (Mp) (40×)

To test the immune modulatory effects of *C. sonorensis* pool feeding using a murine model, adult female midges were allowed to feed on anesthetized 10-week-old female BALB/c mice for 30 min (Fig. 1a). Directly after being exposed, mice showed significant bite site trauma with redness and swelling throughout the abdominal area (Fig. 1b). The underside of the skin showed distinct bite sites and significant hemorrhaging (Fig. 1c). On average, 114 ± 27 midges were determined as fully engorged per mouse. At 3 h post-feeding (hpf), external signs of feeding had dramatically abated and by 8 hpf trauma from midge feeding could not be easily detected. By 1 day post-feeding (dpf), the only external sign of midge feeding was a mild dry skin condition found on several of the mice resembling psoriasis. The majority of the mice had pink healthy skin and exhibited no signs of soreness or distress.

**Fig. 2:**
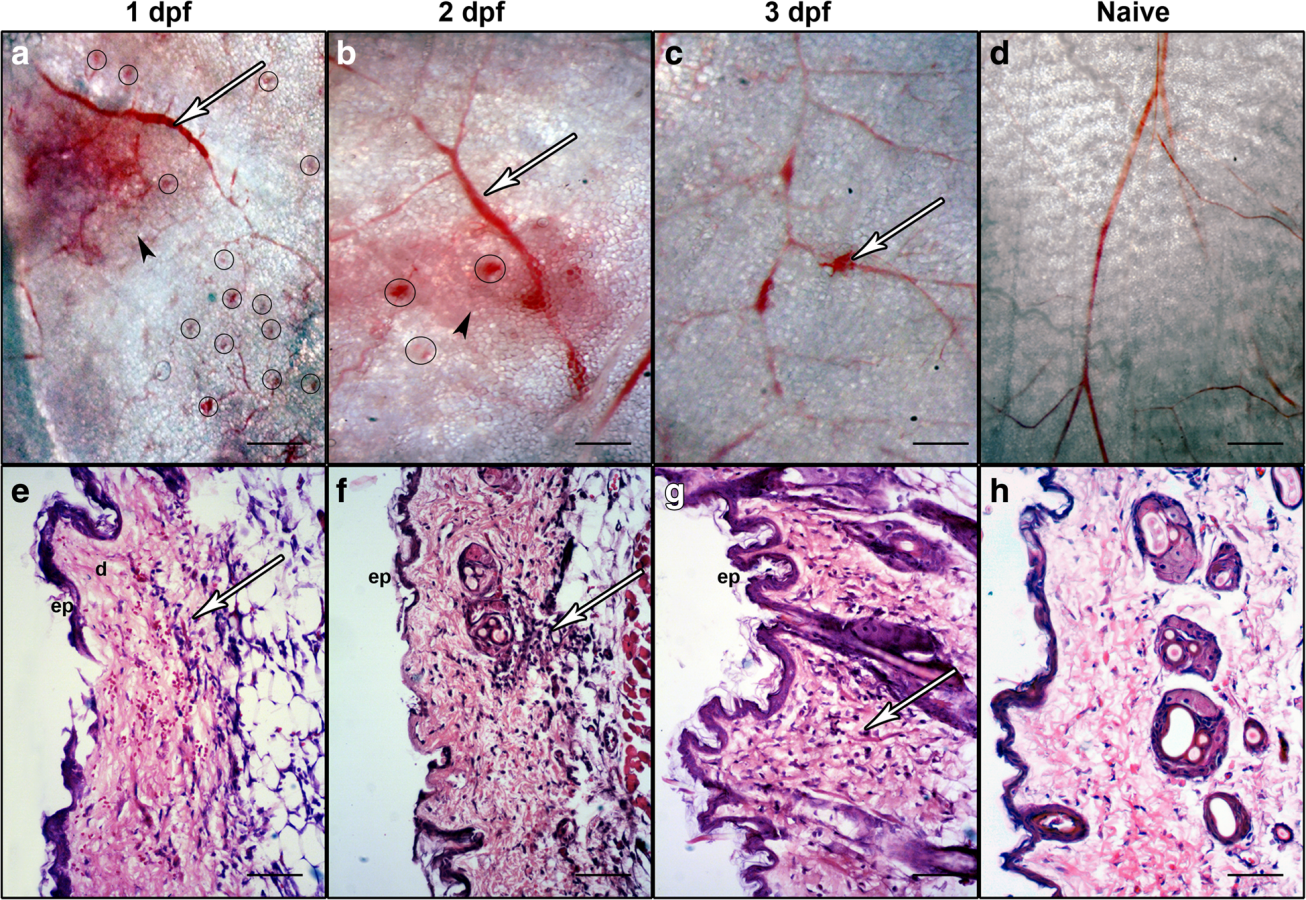
Damage of abdominal dermis in BALB/c mice following feeding by *Culicoides sonorensis*. Representative images at 1–3 days post-feeding (dpf). **a** 1 dpf with bite marks (circles), subcutaneous hematomas (arrowhead), and vasodilation (arrow) 10×. **b** By 2 dpf, subcutaneous hematomas were clearing (arrowhead), vasodilation was still apparent (arrow) and bite sites were diminishing except in areas where a bite had bisected a capillary (circles) 20×. **c** By 3 dpf, hematomas had largely been reabsorbed and vascular damage was restricted to areas directly damaged during feeding (arrow) 20×. **d** Abdominal skin of unexposed, naive mouse 7.5×. **e** H&E staining of micro-thin cross sections of mouse skin at 1 dpf showing damage to epidermis (e) associated with extravasated erythrocytes in the hypodermis (d) (arrow). **f** By 2 dpf, infiltrating leukocytes were seen at bite sites and the majority of extravasated erythrocytes had been cleared (arrow). **g** At 3 dpf, infiltrating leukocyte numbers in focal areas decreased (arrow), but were still more prevalent than that seen in the abdominal skin of naïve mice not exposed to feeding *Culicoides* midges (H). Magnifications of **e**-**h**: 200×; *scale*-*bars*: 500 μm

At 1, 2 and 3 dpf, six mice were sacrificed and the inner surface of abdominal skin examined. Despite lacking external evidence of feeding by 8 hpf, the inner surface of the skin at 1 dpf clearly showed bite sites, large areas of subcutaneous hemorrhage, as well as vascular enlargement (Fig. 2a). Biopsy skin sections showed classical signs of edema as well as a large number of erythrocytes throughout the dermis and hypodermis (Fig. 2e). By 2 dpf, subcutaneous hematomas were in the process of reabsorption, vasodilation was still apparent and bite sites were diminishing (Fig. 2b). Infiltrating leukocytes were seen at bite sites and the majority of extravasated erythrocytes had been cleared (Fig. 2f). By 3 dpf, hematomas had largely been reabsorbed and vascular damage was only visible where biting mouthparts (Fig. 1d, e) had directly bisected capillaries (Fig. 2c) and fewer sites of leukocyte infiltration were visible in sections (Fig. 2g).

**Fig. 3:**
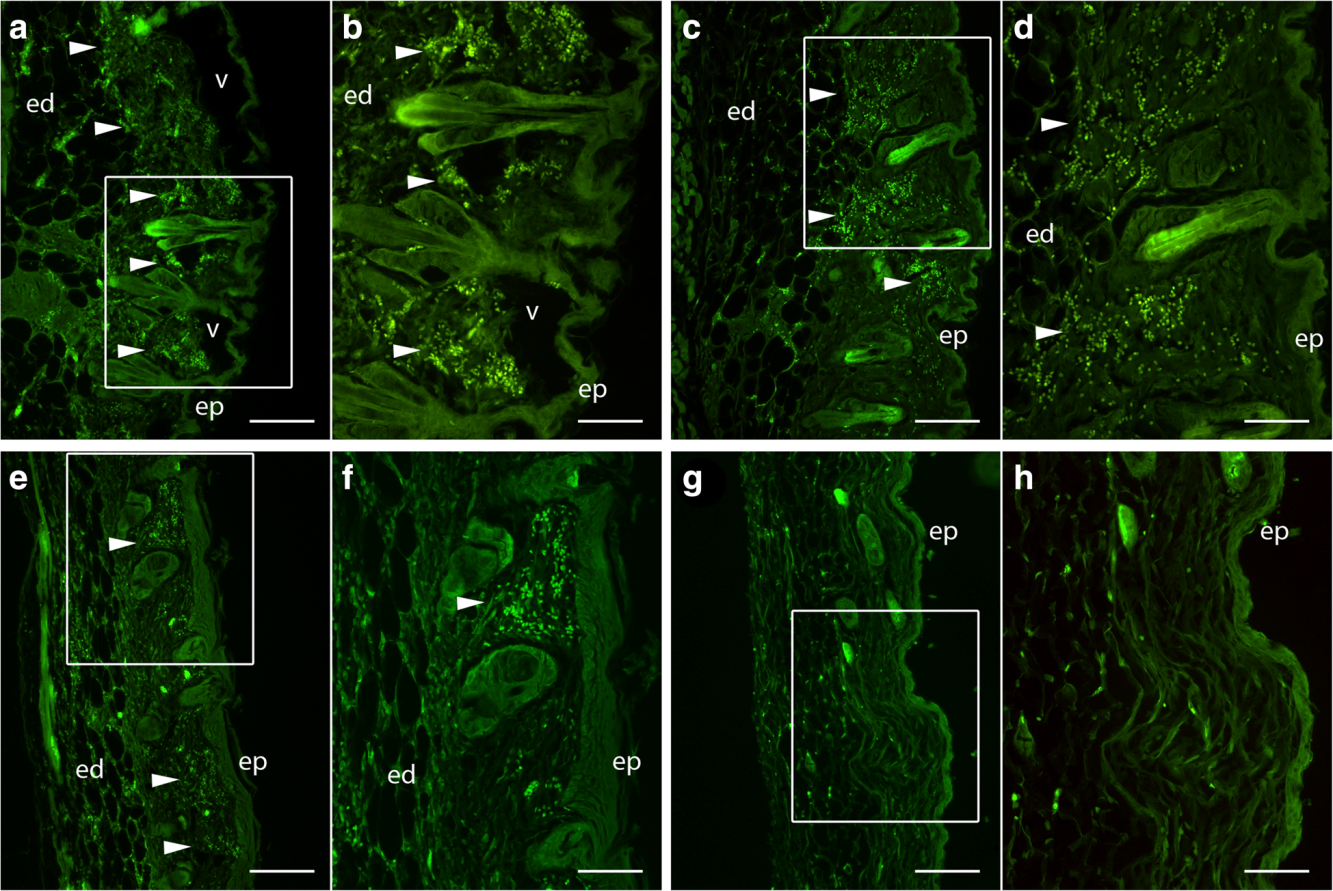
Dermal infiltration of CD16/32+ leukocytes in response to *Culicoides sonorensis* midge feeding. Immunofluorescent staining of micro-thin sections of BALB/c mouse abdominal skin with an anti-CD16/32 FcγIII/II receptor antibody shows infiltrating CD16+/32+ cells in hypodermal spaces beneath midge-exposed epidermis (ep), associated with edema (ed) and fluid filled vesicles (v). Boxed areas in **a**, **c**, **e** and **g** (100×; *scale*-*bars*: 100 μm) reflect regions of higher magnification shown in **b**, **d**, **f** and **h** (200×; *scale*-*bars*: 50 μm). Time points shown are 1-day post-feeding (dpf) (**a**, **b**); 2 dpf (**c**, **d**); 3 dpf (**e**, **f**); compared to naïve control mice with not exposed to midge feeding (**g**, **h**)

Staining with a CD16/CD32 antibody, which recognizes FcγIII/II receptor of a variety of immune cells such as natural killer cells, monocytes, macrophages, dendritic cells, granulocytes, and mast cells showed many foci of infiltrating immune cells into the hypodermis at 1 and 2 dpf (Fig. 3a-d). By 3 dpf, fewer foci of cellular infiltration were visible (Fig. 3e, f), but still clearly more prevalent than control naïve mice (Fig. 3g, h). This suggests the immune response to midge feeding remains in effect in the bite areas on day 3 even after the damage from the *C. sonorensis* bite has been largely repaired as seen in Fig. 2. Autofluorescence of hair shafts as well as murine erythrocytes was seen as has been previously described [44].

### Lymph node responses to *C. sonorensis* feeding

**Fig. 4:**
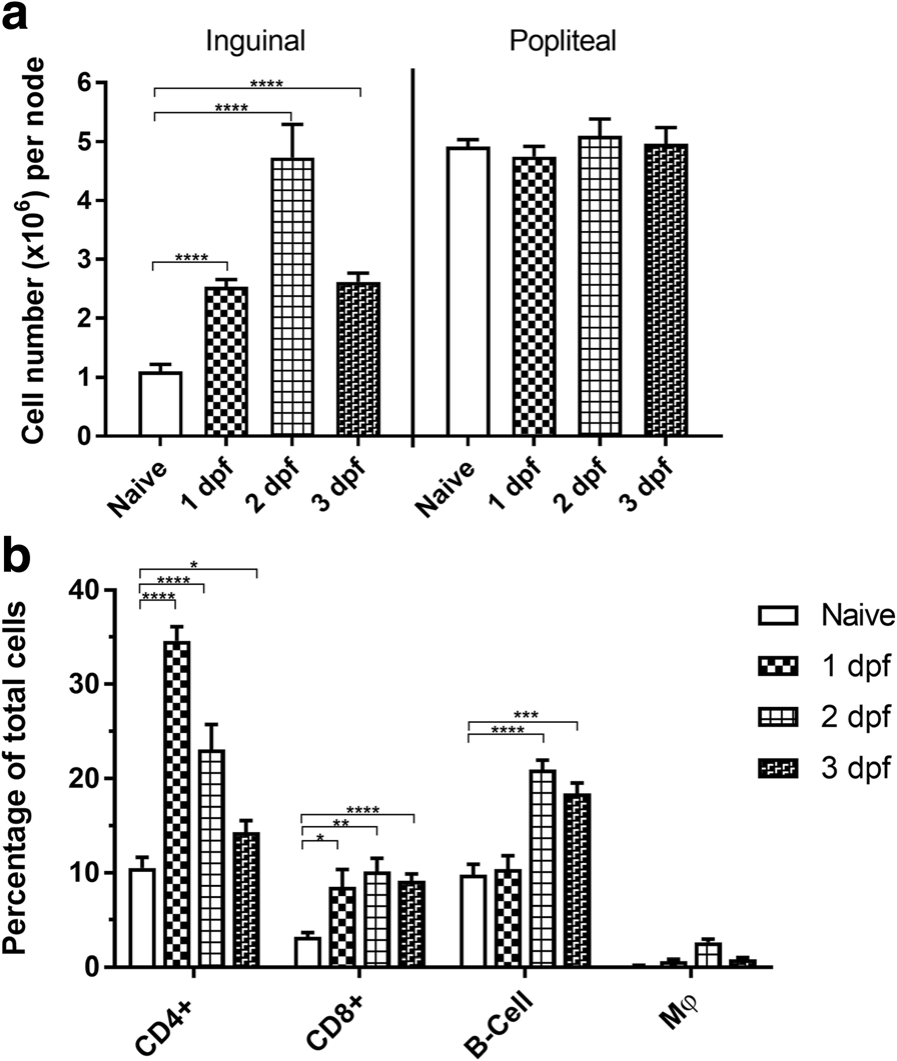
Enlargement of inguinal lymph nodes in response to *Culicoides sonorensis* midge feeding. **a** Manual counts of inguinal and popliteal lymph node cell suspensions from mice harvested at 1, 2 and 3 days post-feeding (dpf) compared to naïve control mice not exposed to feeding midges. **b** Flow cytometric analysis showing the proportions of CD4^+^ cells, CD8^+^ cells, B-cells, and macrophages (Mφ) populations in the mixed inguinal cell populations of mice harvested at 1, 2 and 3 dpf compared to naïve controls. (*P < 0.05, **P < 0.01, ***P < 0.001, ****P < 0.0001; multiple t-tests with two-stage linear step-up procedure of Benjamini et al. [41])

Inguinal and popliteal lymph nodes were examined at 1, 2 and 3 dpf. Node pairs from each animal were made into single cell suspensions and counted manually (Fig. 4a). Inguinal lymph nodes, which drain the abdominal midge feeding region [45], became significantly enlarged by 1 dpf with 2.533 × 10^6^ ± 0.123 cells/node compared to naïve controls at 1.103 × 10^6^ ± 0.121 cells/node (*t*_(10)_ = 8.306, *P* < 0.0001). By 2 dpf, the inguinal nodes were clearly enlarged upon necropsy with 4.733 × 10^6^ ± 0.564 cells/node compared to naïve controls (*t*_(10)_ = 6.295, *P* < 0.0001), and by 3 dpf with 2.617 × 10^6^ ± 0.149 cells/node (*t*_(10)_ = 7.891, *P* < 0.0001). Popliteal lymph nodes, distal to the bite areas, showed no significant difference between midge-exposed and naïve control mice suggesting changes in the inguinal nodes were the result of localized draining from the bite sites and not a systemic effect.

To further categorize node enlargement, inguinal node cell suspensions were labeled with antibody pairs specific for CD4^+^ and CD8^+^ T-cells, B-cells and macrophage cells. Suspensions were analyzed using FSC/SSC flow cytometry gating and reported as a percentage of the total cells present (Fig. 4b). The proportion of CD4^+^ cells of the total inguinal node cells was elevated significantly on day 1 with 34.62 ± 1.515% (*t*_(10)_ = 12.68, *P* < 0.0001), day 2 with 23.12 ± 2.59% (*t*_(10)_ = 4.451, *P* = 0.0012), and day 3 with 14.32 ± 1.243% (*t*_(10)_ = 2.254, *P* = 0.0478) compared to naïve unexposed mice (10.5 ± 1.151%). The proportion of CD8^+^ cells of the total number of cells was elevated significantly on day 1 with 8.5 ± 1.838% (*t*_(10)_ = 2.807, *P* = 0.0186), day 2 with 10.13 ± 1.411% (*t*_(10)_ = 4.684, *P* = 0.0008), and day 3 with 9.133 ± 0.737% (*t*_(10)_ = 6.858, *P* < 0.0001) compared to naïve unexposed mice (3.183 ± 0.4578%). The proportion of B cells of the total number of cells was elevated significantly on day 2 with 20.98 ± 1.006% (*t*_(10)_ = 7.296, *P* < 0.0001), and on day 3 with 18.43 ± 1.133% (*t*_(10)_ = 5.34, *P* = 0.0003) compared to naïve unexposed mice (9.767 ± 1.162%). The proportion of macrophage cells of the total number of cells was elevated significantly on day 1 with 0.633 ± 0.173% (*t*_(10)_ = 2.54, *P* = 0.0294, on day 2 with 2.617 ± 0.331% (*t*_(10)_ = 7.296, *P* < 0.0001), and on day 3 with 0.833 ± 0.1358% (*t*_(10)_ = 4.59, *P* = 0.0009) compared to naïve unexposed mice (0.183 ± 0.040%).

### Cell-mediated immune responses to *C. sonorensis* feeding

**Fig. 5:**
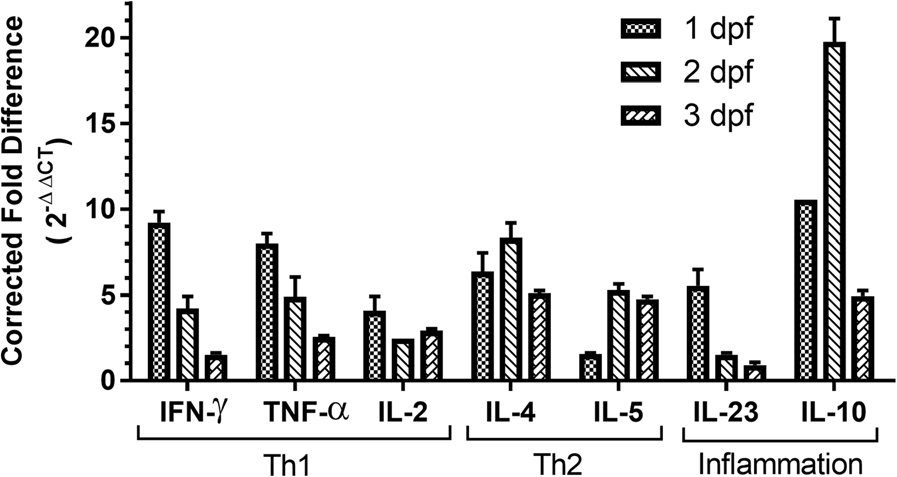
Cytokine expression upregulation in inguinal lymph nodes in response to *Culicoides sonorensis* midge feeding. Quantitative RT-PCR was used to measure cytokine expression in inguinal lymph node cells at 1, 2 and 3 days post-feeding (dpf) by *C. sonorensis*. Increased expression levels (y-axis) are reported as a corrected fold difference (2^-ΔΔC^_T_) compared to naïve controls (basal level). Beta actin was used as an internal control

Proliferation of the CD4^+^ cell population in inguinal lymph nodes suggested a Th-mediated cellular response was elicited by the *C. sonorensis* exposure. To determine whether this was a pro-inflammatory Th1 or Th2 response, select cytokine expression in the inguinal lymph node cells was examined by quantitative RT-PCR at 1, 2 and 3 dpf (Fig. 5). At 1 dpf, expression of Th1 mediators IFN-γ, TNF-α and IL-2 increased (9.21 ± 0.9-fold, 8.02 ± 0.7-fold, and 4.09 ± 1.1-fold, respectively), indicating a potent initial pro-inflammatory response which decreased at 2 and 3 dpf. Expression levels of the Th2 cytokines IL-4 and IL-5 were 6.37 ± 1.5 and 1.57 ± 0.7-fold, respectively, higher than naïve controls at 1 dpf and increased to 8.32 ± 1.2 and 5.29 ±0.51-fold by 2 dpf. By 3 dpf, the levels of these cytokines had decreased slightly from their 2 dpf peak but were still significantly higher than basal expression. The Th17 pro-inflammatory mediator IL-23 showed a 5.54 ± 1.34-fold increase by 1 dpf, but the overall levels of this cytokine decreased to nearly basal levels by 2 and 3 dpf. The anti-inflammatory cytokine IL-10, had the highest expression upregulation of any cytokine measured, increasing over 10-fold over the naïve control by 1 dpf and nearly 20-fold by 2 dpf and likely contributed to the Th2 skewed response. Increased expression levels were calculated as a corrected fold difference (2^-ΔΔC^_T_) compared to naïve controls [43].

### Mast cell degranulation in response to *C. sonorensis* feeding

**Fig. 6:**
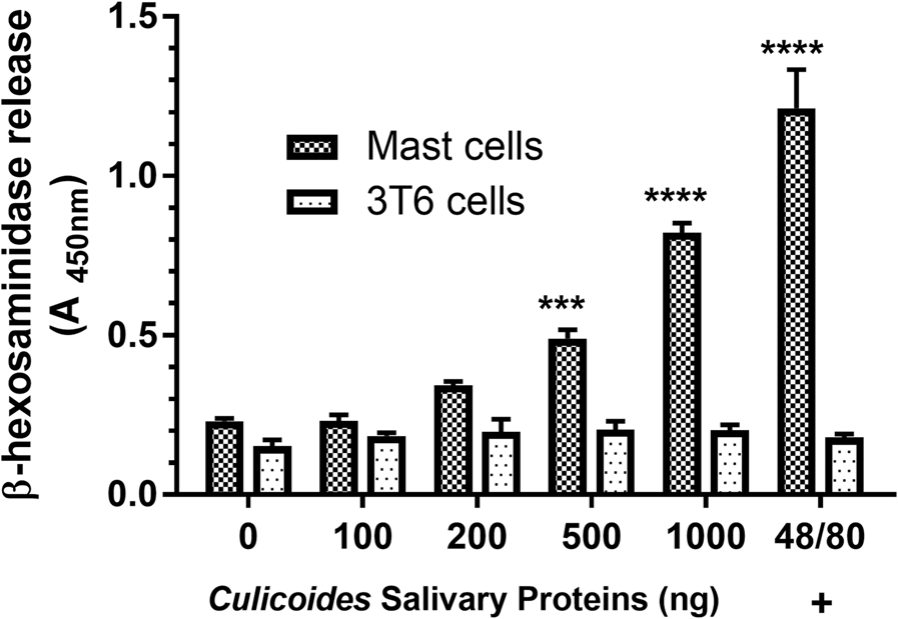
Dose-dependent mast cell degranulation in response to *Culicoides sonorensis* salivary proteins. Connective tissue mast cells (CTMCs) or 3T6 fibroblast cells were exposed to *C. sonorensis* salivary proteins (100–1000 ng/ml) or the potent chemical mast cell activator compound 48/80 for 1 h after which *β*-hexosaminidase release was quantified. (***P < 0.001, ****P < 0.0001; two-way ANOVA with Sidak’s multiple comparisons test)

To determine the effects of *C. sonorensis* saliva in mast cell activation/degranulation and the role degranulation played in the Th2-mediated responses to feeding, both *in vitro* and *in vivo* studies were conducted. *In vitro*, a connective tissue mast cell line derived *ex vivo* and driven to differentiate, underwent degranulation in the presence of *Culicoides* salivary proteins in a dose-dependent manner (Fig. 6).

**Fig. 7:**
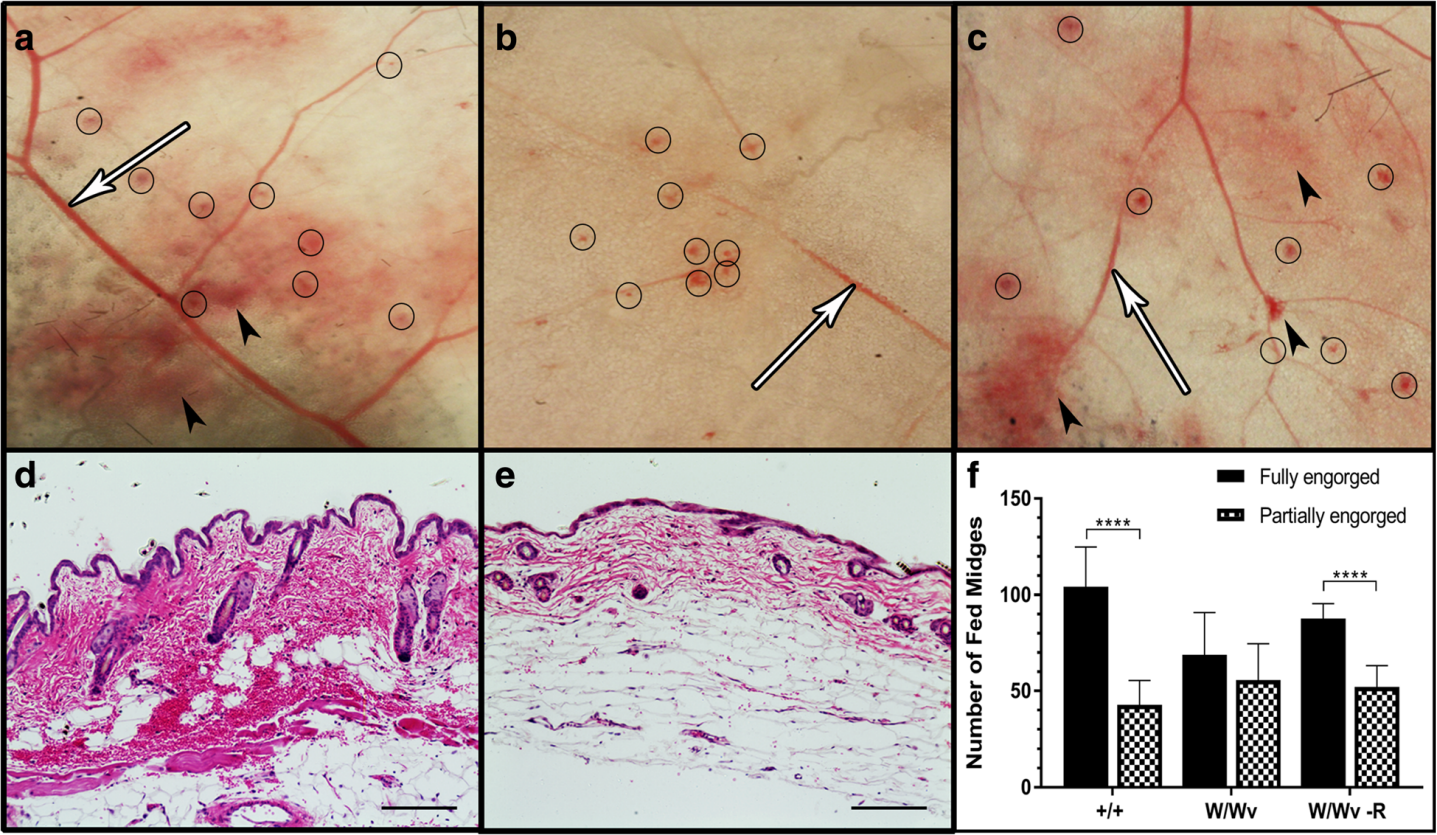
Mast cell activation critical for innate immune response to *Culicoides sonorensis* midge feeding. Ten-week-old (**a**) wild type mast cell-sufficient (WBB6F1-^+/+^), (**b**) mast cell-deficient (WBB6F1-^W/Wv^) and (**c**) mass cell-reconstituted (WBB6F1-^W/Wv^ -R) mice 3 h post-exposure to feeding *C. sonorensis*. Midge bites (circles), areas of extensive subcutaneous hemorrhaging (black arrowheads) and vasodilated dermal capillaries (white arrows) are shown most prominently in both the wild type +/+ and CTMC reconstituted W/Wv-R mice. H&E staining of micro-thin sections of abdominal skin showing extensive erythrocytes released in the dermis and hypodermis of wild type +/+ mice (**d**) compared to deficient W/Wv (**e**) mice. **f** Manual counts of fed *C. sonorensis* females (fully *vs* partially) between wild type mast cell-sufficient (+/+), mast cell-deficient (W/Wv), and CTMC-reconstituted (W/WV-R) mouse groups. (Magnifications: **a**-**c** 100×, *scale*-*bars*: 100 μm; **d** and **e** 200×, *scale*-*bars*: 50 μm). (****P < 0.0001; multiple t-tests with two-stage linear step-up procedure [41])

*In vivo*, *C. sonorensis* midges were allowed to feed on wild type mast cell-sufficient mice (WBB6F1-^+/+^), mast cell-deficient mice (WBB6F1-^W/Wv^), and CTMC-reconstituted mice (WBB6F1-^W/Wv^ -R). Microscopic examination of the underside of the skin at 3 hpf showed both wild type and CTMC-reconstituted mice had extensive subcutaneous hemorrhages spread throughout the dermis and hypodermis with enlarged, leaky vascular networks (Fig. 7a, c). Conversely, the mast cell-deficient mice had less visible subcutaneous hemorrhage when examined under the microscope and the vascular network had minimal areas of vasodilation, even with multiple bite sites and lesions clearly visible adjacent to dermal capillaries (Fig. 7b). Cross-sections of the wild type mice skin (Fig. 7d) showed large numbers of erythrocytes spread throughout the hypodermis accounting for the visible subcutaneous hemorrhage noted in the initial inspection. The skin of mast cell-deficient mice (Fig. 7e) appeared to have extensive remodeling of the dermis which is common with swelling, and sporadic extravasated erythrocytes could be seen; however, sections lacked obvious signs of extensive hemorrhage seen in wild type and CTMC-reconstituted mice.

In assessing blood-feeding efficiency, there was no significant difference in the average total number of fed *C. sonorensis* females (fully and partially combined) between the groups, with 147 for wild type, 124 for mast cell-deficient, and 139 for CTMC-reconstituted mice (Fig. 7f). There were, however, significantly higher numbers of fully engorged midges compared to partially fed midges in the wild type (*t*_(10)_ = 6.233, *P* < 0.0001) and CTMC-reconstituted groups (*t*_(10)_ = 6.408, *P* < 0.0001), but not for the mast cell-deficient mice (*t*_(10)_ = 1.113, *P* = 0.292).

**Fig. 8:**
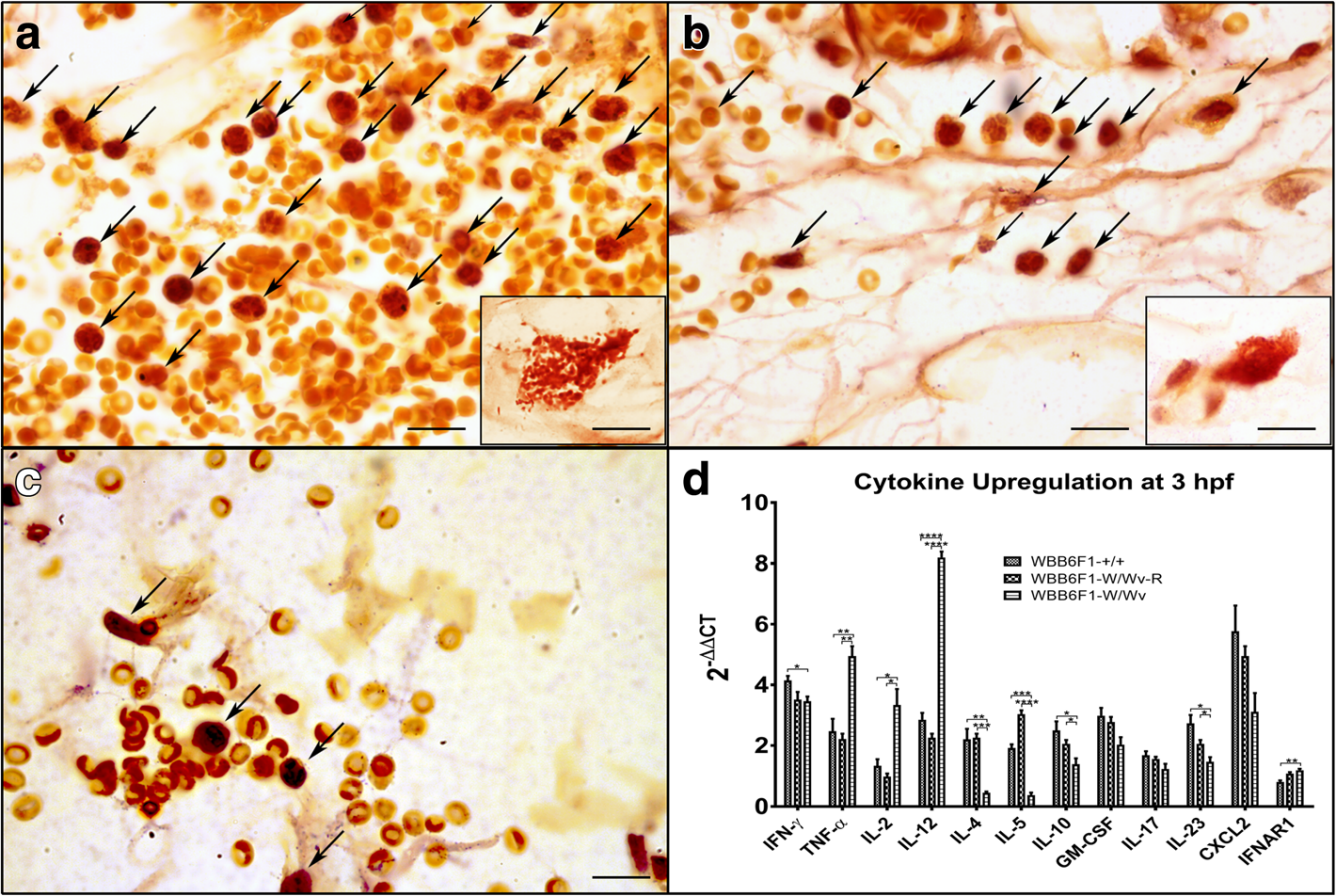
Granulocyte infiltration and cytokine upregulation in response to *Culicoides sonorensis* midge feeding is mast cell dependent. Micro-thin cross abdominal skin sections from (**a**) 10-week-old wild type mast cell-sufficient (WBB6F1-^+/+^) mice, (**b**) CTMC-reconstituted (WBB6F1-^W/Wv^ -R) mice or (**c**) mast cell-deficient (WBB6F1-^W/Wv^) mice at 3 h post-feeding (hpf) by *C. sonorensis* stained for chloroacetate esterase activity indicative of activated granulocytes. Insets show degranulating mast cells. Arrows showing infiltrating granulocytes, primarily of neutrophil lineage, were abundant in abdominal areas associated with midge feeding in wild type and mast cell-reconstituted mice. (Magnifications: 1000×, *scale*-*bars*: 10 μm). **d** Quantitative RT-PCR measuring cytokine expression in inguinal lymph node cells of mast cell-deficient (W/Wv), mast cell-sufficient (+/+), and CTMC-reconstituted (W/Wv-R) mouse strains at 3 hpf. Increased expression levels (y-axis) are reported as a corrected fold difference (2^-ΔΔC^_T_) compared to naïve controls (basal level). Beta actin was used as an internal control. Additionally, wild type (+/+) and reconstituted (W/Wv-R) groups were compared to the mast cell-deficient (W/Wv) group (*P < 0.05, **P < 0.01, ***P < 0.001, ****P < 0.0001; Holm-Sidak t-test for multiple comparisons between the groups within each cytokine)

Sections of skin from wild type mast cell-sufficient, mast cell-deficient and CTMC-reconstituted mice were stained for chloroacetate esterase to detect mast cell activation and granulocyte infiltration. Both the wild type and CTMC-reconstituted mice had increased infiltrating granulocytes, primarily neutrophils often embedded with extravasated red blood cells, throughout the dermis (Fig. 8a, b, arrows). Degranulating mast cells could be easily identified (insets). In contrast, the mast cell-deficient line had markedly lower numbers of infiltrating granulocytes (Fig. 8c, arrows) which were primarily co-localized with extravasated erythrocytes in the hypodermis.

To investigate early changes in the bite site environment that may influence the incipient immune responses, RNA expression of select cytokines was measured in abdominal skin samples taken from the three genetic mouse lines at 3 hpf (Fig. 8d) compared to unexposed naïve mice. Expression levels of the pro-inflammatory cytokines IL-2 and IL-12, along with TNF-α, were all significantly elevated in the mast cell-deficient line compared to the wild type and CTMC-reconstituted lines. This upregulation was coupled with a relative decrease in the expression levels of Th-2 cytokines IL-4, IL-5 and IL-10 in the mast cell-deficient line. The relative expression of Chemokine-C-X-C motif ligand 2 (CXCL2) was also significantly lower in the mast cell-deficient mice. CXCL2 (also known as macrophage inflammatory protein 2 or MIP-2) is a chemotactic factor released by macrophages, mast cells and CD4^+^ cells that induces the infiltration of granulocytes from the bloodstream to wounds and could account for the fewer observed granulocytes seen in the chloroacetate esterase staining of the mast cell-deficient mice skin. Increased expression levels were calculated as a corrected fold difference (2^-ΔΔC^_T_) compared to naïve controls [43]. Additionally wild type and CTMC-Reconstituted groups were compared to the mast cell deficient group within each cytokine by Holm-Sidak t-test for multiple comparisons.

## Discussion

Pool-feeding hematophagous *Culicoides* midges are important agricultural pests and arboviral vectors, yet little information has been reported to date regarding the physiological effects of, and immune responses elicited by midge feeding. Here, using a mouse model and colonized *C. sonorensis* midges, feeding induced clearly visible subcutaneous hemorrhages in the dermis at the wounding site. Cutaneous ulcers/papules resolved almost entirely by 8 hpf leaving no evidence of feeding, unlike the persisting papules reported for guinea pigs at 5 or more days after midge feeding [46]. Mast cell degranulation occurs when allergens are introduced, but also in response to injury [47]. Comparisons of mast cell-deficient to wild type mice suggests dermal capillary vasodilation and feeding hematomas are mediated, in part by the wound damage caused by the midge’s mouthparts piercing dermal capillaries, and in part through saliva-mediated activation and subsequent degranulation of connective tissue mast cells. Components found in the saliva of other hematophagous insects have also been shown to directly induce mast cell degranulation [48]. Once activated, mast cells release preformed mediators of inflammation including the biogenic amine histamine, lysosomal enzymes such as β-hexosaminidase, and serine proteases such as tryptase β-hexosaminidase, and in mice serotonin [49, 50]. For quick feeding pool feeders, these responses are critical for rapidly inducing the edema and erythrocyte release required for engorgement. It is interesting to note that the only group where numbers of fully engorged midges were not significantly higher than partially fed was the mast cell-deficient mice. This supports the importance of mast cell-related physiological effects for the ability of *Culicoides* to feed to repletion.

In addition to these short-term effects, *C. sonorensis* feeding also had longer lasting immune modulatory effects. At the cellular level, CD16/32+ leukocytes infiltrating the dermis were much more abundant at 1, 2 and 3 dpf compared to naïve controls, with the peak occurring on day 2. Significant hyperplasia of draining inguinal lymph nodes indicates this leukocyte homing is activated in multiple tissues. The lack of hyperplasia in popliteal lymph nodes suggests a local, rather than systemic effect, as they do not drain abdominal skin and lymphatics from inguinal nodes do not drain to popliteal nodes. Decreased numbers of infiltrating neutrophils in mast cell-deficient mice suggests mast cell activation plays a critical role in this initial cellular response. Histamine release during mast cell activation is of particular importance here because in addition to being a potent chemotactic agent for the recruitment of other granulocytes (including mast cells) into the affected area, it also serves to activate nearby dendritic cells leading them to increase their production of IL-10 [51]. After the degranulation process is underway, connective tissue mast cells also rapidly synthesize and secrete several lipid-derived eicosanoids (thromboxane, platelet activating factor, and leukotrienes) involved in vascular regulation as well as a number of cytokines including the Th2 mediators IL-4 and IL-5.

Analyses of both inguinal node cell populations and cytokine expression profiles clearly showed a Th-mediated cellular response pattern. One likely factor in the overall decline of pro-inflammatory Th1 mediators IL-2, IFN-γ, and TNF-α by day 3 was the 20-fold increase in IL-10 on day 2; a potent anti-inflammatory cytokine produced by multiple cell types including CD4^+^ lymphocytes and activated mast cells. The role of activated mast cells in this response was corroborated by results of the mast cell *in vivo* experiment where mast cell-deficient mice showed significantly higher levels of pro-inflammatory cytokine expression (IL-2, IL-12 and TNF-α) and significantly lower levels of the anti-inflammatory cytokines (IL-4, IL-5, and IL-10) compared to wild type and CTMC-reconstituted mice. This pro-inflammatory response, specifically the increased swelling, redness and raised papules, was still evident in the mast cell-deficient mice at 3 hpf, but nearly absent in the wild type mice. Based on these results, mast cells contribute to the immediate response to *Culicoides* feeding, as well as to the magnitude and perpetuation of Th2-biased systemic responses, which would have possibly led to an adaptive allergic response such as that seen in IBH horses, had the stimuli continued. It is important to note that since this model used laboratory animals naïve to midge feeding, it may more accurately reflect local responses in initial exposures to midge feeding, and not account for the possibly amplified reaction of those animals pre-sensitized to midge bites such as animals frequently exposed to the swarm feeding of midges. It is expected that compared to our mouse model, these animals would have increased numbers of mast cells in tissues repeatedly exposed to midge feeding *via* mast cell or progenitor migration, or *via* resident mast cell precursor proliferation [47]. This would result in increased degranulation and inflammation, increased CD16/32+ cell populations in dermal tissues, and likely slower skin healing.

The immune response pattern elicited by *Culicoides* feeding and saliva deposition fits well into the current knowledge of the orbivirus infection model, specifically BTV, EHDV and AHS. It is understood that mice serve only as a limited model for ruminants, as they are not exposed naturally over time to midge feeding and therefore do not have a memory response that might be expected in pastured animals. Additionally, orbiviruses have a tropism for γδ T-cells [33], a major circulating lymphocyte population in ruminants, thus an important contributor to viral replication, whereas mice have a low level of circulating γδ T cells [52]. That said, our results show that *Culicoides* saliva does not just act as a viral carrier, rather it potentiates a Th2-mediated response which leads to a rapid infiltration and proliferation of CD16/32+ lymphocytes, macrophages, neutrophils and dendritic cells, known infection targets of BTV [33, 34, 53], EHDV [54] and AHS [55]. In response to feeding, infected lymphocytes drain to nearby lymph nodes which become hyperplastic due to the saliva. As these hemorrhagic viruses have a tropism for lymphatic tissues, hyperplastic lymph nodes become centers of viral replication, rapidly spreading infection throughout the animal *via* the lymphatic system while impairing B-cell activation and antibody production [56]. Additionally, these viruses preferentially infect microvascular endothelial cells [34, 53, 55] which are directly exposed immediately through mechanical disruption of dermal capillaries by biting mouthparts, as well as indirectly through the vasodilation response to midge saliva. Subsequent viral shedding and efficient dissemination *via* the circulatory system would be expected.

## Conclusions

As demonstrated in this mouse model, immune responses, physiological changes, and recruitment of cells known to be orbivirus infection targets directly to bite sites where viral particles are being deposited during blood-feeding, may help explain the ability of a single infected midge to transmit BTV to naïve sheep with 80–100% efficiency [22]. *Culicoides* feed quickly (1–2 min), are extremely minute (2 mm), and therefore, deliver proportionally minute amounts of saliva during feeding. However, since *Culicoides* are swarm feeders, thousands of midges may descend upon an animal to feed, thereby cumulatively introducing saliva over a wide area of the skin, priming it for rapid infection by the minuscule viral inoculum delivered by the bite of a single infected midge. Mammalian responses to midge feeding clearly contribute to the efficiency of this insect species as a vector for orbiviruses which have evolved to exploit these immune pathways to facilitate rapid localized initial infections and subsequent systemic dissemination.
